# Takeaway food consumption and its associations with diet quality and abdominal obesity: a cross-sectional study of young adults

**DOI:** 10.1186/1479-5868-6-29

**Published:** 2009-05-28

**Authors:** Kylie J Smith, Sarah A McNaughton, Seana L Gall, Leigh Blizzard, Terence Dwyer, Alison J Venn

**Affiliations:** 1 Menzies Research Institute, University of Tasmania, Hobart Tasmania 7000, Australia; 2 Centre for Physical Activity & Nutrition Research, Deakin University, Burwood Victoria 3125, Australia; 3 Murdoch Childrens Research Institute, Royal Children's Hospital, Parkville Victoria 3052, Australia

## Abstract

**Background:**

Few studies have investigated the associations of takeaway food consumption with overall diet quality and abdominal obesity. Young adults are high consumers of takeaway food so we aimed to examine these associations in a national study of young Australian adults.

**Methods:**

A national sample of 1,277 men and 1,585 women aged 26–36 completed a self-administered questionnaire on demographic and lifestyle factors, a 127 item food frequency questionnaire, usual daily frequency of fruit and vegetable consumption and usual weekly frequency of takeaway food consumption. Dietary intake was compared with the dietary recommendations from the Australian Guide to Healthy Eating. Waist circumference was measured for 1,065 men and 1,129 women. Moderate abdominal obesity was defined as ≥ 94 cm for men and ≥ 80 cm for women. Prevalence ratios (PR) were calculated using log binomial regression. Takeaway food consumption was dichotomised, with once a week or less as the reference group.

**Results:**

Consumption of takeaway food twice a week or more was reported by more men (37.9%) than women (17.7%, P < 0.001). Compared with those eating takeaway once a week or less, men eating takeaway twice a week or more were significantly more likely to be single, younger, current smokers and spend more time watching TV and sitting, whereas women were more likely to be in the workforce and spend more time watching TV and sitting. Participants eating takeaway food at least twice a week were less likely (P < 0.05) to meet the dietary recommendation for vegetables, fruit, dairy, extra foods, breads and cereals (men only), lean meat and alternatives (women only) and overall met significantly fewer dietary recommendations (P < 0.001). After adjusting for confounding variables (age, leisure time physical activity, TV viewing and employment status), consuming takeaway food twice a week or more was associated with a 31% higher prevalence of moderate abdominal obesity in men (PR: 1.31; 95% CI: 1.07, 1.61) and a 25% higher prevalence in women (PR: 1.25; 95% CI: 1.04, 1.50).

**Conclusion:**

Eating takeaway food twice a week or more was associated with poorer diet quality and a higher prevalence of moderate abdominal obesity in young men and women.

## Background

Consuming takeaway or fast food is becoming more prevalent in Australia [1] and around the world [2,3]. Although there are no standard definitions, fast food is the term used in North America and typically includes food that can be obtained quickly such as burgers, fries, pizza and fried chicken. Takeaway is the common term used in Australia and includes fast food and other "take out" meal options such as Thai and Indian food. The majority of previous studies were conducted in the USA and focused on fast food. Although it is known that takeaway and fast food consumption is higher in younger age groups than older age groups [4-9] and consumption of fast food has been shown to increase from adolescence to young adulthood [10], there is little research focusing on the correlates of takeaway food consumption in young adults. Furthermore, the socio-economic and lifestyle characteristics of individuals eating takeaway food have not previously been reported separately for men and women [4,6-9]. It is important to see if the characteristics associated with high takeaway food consumption differ between men and women and to identify groups that consume high levels of takeaway food.

A high frequency of takeaway and fast food consumption has been linked to poorer diet quality including a lower intake of vegetables [5,7,8,11], wholegrains [7], low fat dairy [7] and fruit [7,9,11], a higher intake of total fat and saturated fat [8,11], sodium [11] and non-diet carbonated soft drinks [4]. Although these studies have shown an association between takeaway and fast food frequency and individual foods, food groups or nutrients, only one previous study from Spain in 1999 measured overall diet quality [9]. However, in this study, the fast food variable included only four items (hamburgers, cheeseburgers, Big Macs and French fries [9]) and excluded other common forms of takeaway food such as pizza, fried chicken, Indian, Chinese and Thai food.

In addition to poorer diet quality, an association between takeaway and fast food consumption and body weight has been reported. The Coronary Artery Risk Development in Young Adults (CARDIA) study in the USA found participants who ate fast food more than twice a week at baseline in 1985 and at the 15 year follow-up had gained an extra 4.5 kg compared with participants who ate fast food less than once a week at both time points [7]. In Spain, participants eating fast food more than once per week had an increased likelihood of being obese (OR 1.29; P = 0.057) compared with non-consumers [9]. In an Australian study in 1996, women eating takeaway once per week were 15% less likely to maintain their weight over a four year period compared with women who ate takeaway never or no more than once per month [12].

Most studies examining associations of fast food or takeaway food consumption and obesity have used body mass index (BMI) as a measure of obesity. Waist circumference is thought to be a better indicator of cardiovascular disease and type 2 diabetes risk than BMI [13,14] as fat distributed around the waist is more harmful than overall obesity. In addition, young adults with high muscle mass might be misclassified as being overweight when using BMI, though the proportion misclassified is not known. One previous study investigating Australian adults living in rural areas during 2001–2003 found no association between high takeaway food consumption and abdominal obesity [15] when using waist circumference as a continuous variable. The authors did not report their findings using recommended cut points for waist circumference to define obesity [16].

The aims of this cross-sectional study of young adults were to examine the socio-economic and lifestyle factors associated with takeaway food consumption in men and women; and to examine associations of takeaway food consumption with diet quality and abdominal obesity.

## Methods

### Participants

The Childhood Determinants of Adult Health (CDAH) Study is a follow-up of children who participated in the 1985 Australian Schools Health and Fitness Survey (ASHFS), a nationally representative study of 8,498 children aged 7–15 years [17].

During 2001–2002 participants were traced through electoral rolls, telephone directories, the National Death Index and contact with class mates. Of the 6,840 (80%) participants successfully traced, 5,170 (61%) were enrolled in the CDAH study and invited to complete questionnaires and attend one of 34 study clinics around Australia for physical measurements. The clinics were held in each state and territory of Australia during 2004–2006, when the participants were aged 26–36 years. Clinics involved a range of physical assessments including anthropometric measurements. In total, questionnaires were completed by 2,881 participants, and 2,410 attended study clinics. The number of participants attending clinics was lower than those enrolled in the CDAH study largely due to the burden of attending the clinic (approximately three hours of testing) and the distance needed to travel.

The study was approved by the Southern Tasmania Health and Medical Research Ethics Committee and all participants gave informed consent.

### Dietary Assessment

Food intakes and habits were measured using a 127 item food frequency questionnaire (FFQ) and a food habits questionnaire (FHQ). The FFQ asked for the average number of times each food and beverage was consumed over the previous twelve months. For each item participants were asked to choose one of nine response options ranging from "never or less than once a month" to "six or more times per day". Daily equivalents were calculated for each FFQ item, assuming one serve was consumed at each eating occasion [18,19]. The mid value was used when the response option included a range of values and missing items were given a value of zero. The FFQ was a modified version of one previously used in the 1995 National Nutrition Survey [19-22] and was based on an existing FFQ developed for Australian populations [23].

The FHQ included questions on takeaway food consumption, daily fruit and vegetable consumption, and frequency of trimming fat from meat. The takeaway food question asked "How many times per week would you usually eat hot takeaway meals (e.g. pizza, burgers, fried or roast chicken, Chinese/Indian/Thai takeaway)". Participants could choose one of five answers ranging from "I don't eat takeaway" to "6–7 meals per week". For analysis, the answers were dichotomised to less than twice per week or twice a week or more as there were small numbers in the lowest and the two higher frequency groups. To assess its validity, responses to the takeaway food question were compared with reported consumption of foods in the FFQ that are commonly eaten as takeaway foods (fried fish, meat pie/sausage roll/other savoury pasties, pizza, hamburger, hot chips/roast potato/potato wedges).

Daily fruit and vegetable consumption came from the two short questions: "how many serves of fruit/vegetables (excluding potatoes) do you usually eat each day". Examples of serving sizes were given and the response options were "I don't eat this food", "1 serve or less", "2–3 serves", "4–5 serves" or "6 or more serves". These short questions have been used in previous studies [19,24] and have been shown to be valid measures for fruit and vegetable intake [25].

Questions from the FHQ and FFQ were used to determine if participants were complying with sex and age-specific recommendations in the Australian Guide to Healthy Eating (AGHE) [26]. The AGHE has been developed to encourage the public to adopt healthy eating patterns by highlighting the foods that help meet nutrient recommendations and provides two recommended patterns of eating. The recommended eating pattern used in this analysis is the most commonly used and is consistent with public health messages that promote consumption of five servings of vegetables and two servings of fruit per day [27]. This eating pattern is also the more conservative of the two for vegetables, fruits and dairy for men. The AGHE recommends adults consume the following number of serves from the five food groups each day: two servings of fruit, five servings of vegetables, two servings of dairy, one serving of lean meat or alternatives and six to twelve servings of breads and cereals for men and four to nine servings of breads and cereals for women.

Foods that do not fit into the five food groups are "extra" foods and are high in fat, salt and sugars and provide very few essential nutrients [26]. The AGHE recommends that these foods be eaten in small amounts. Examples of extra foods include ice cream, cream, cakes, sweet pies, desserts, sweet biscuits, chocolate biscuits, savoury pastry, pizza, hamburgers, hot chips, fried fish, chocolate, other confectionary, crisps, dressings, mayonnaise, jam, creamy dips, fruit drink, cordial, soft drink and all alcohol. The guidelines recommend limiting the number of "extra" foods to no more than three servings per day for men and no more than two and a half servings per day for women. For analysis the extra foods variable was created excluding the takeaway food items (hamburgers, pizza, hot chips, fried fish and savoury pastry), so that takeaway food items could be distinguished separately.

For comparison with the AGHE, information on daily servings of fruit and vegetables came from the short questions in the FHQ. Daily serves of breads and cereals, dairy, lean meat and alternatives and extra foods were obtained from summing daily equivalents calculated from the FFQ (see Appendix 1 for items included in each food group). For breads and cereals, the lowest recommended value was used, and for extra foods participants not exceeding the upper limit were classified as meeting the recommendation. In line with the Dietary Guidelines for Australian Adults [28] high fat meats were not included in the meat and alternatives food group. However, some meat items that would be considered lean if the visible fat was removed were included as lean meats (see Appendix 1) if participants reported in the FHQ that they "usually" trimmed the fat from their meat either before or after cooking. The analysis of fruit and vegetable intake was also repeated using items from the FFQ.

### Anthropometric measurements

For the anthropometric measurements, participants were standing and dressed in light clothing without shoes. All measurements were made by trained staff. Waist circumference was measured in triplicate over light clothing at the narrowest point between the lower costal border and the iliac crest, at the end of normal expiration. Measurements were taken using a Lufkin steel (non-stretch) tape measure and were recorded to the nearest 0.5 cm. Moderate abdominal obesity was defined as ≥ 94 cm for men and ≥ 80 cm for women. These cut points were defined by the World Health Organization and are associated with an increased risk of metabolic complications associated with abdominal obesity [16].

Body weight was measured using a Heine portable scale (Heine, Dover, NH, USA) and recorded to the nearest 0.1 kg. Height was measured using a portable Leicester stadiometer (Invicta, Leicester, UK) and recorded to the nearest 0.1 cm. BMI (kg/m^2^) was calculated from height and weight.

### Covariates

Demographic variables included age, sex, education (classified as school only, vocational, university), employment status (working versus not in the workforce) and marital status (married or living as married versus other). Smoking was classified based on self report as never, former or current smoker.

The long version of the International Physical Activity Questionnaire (IPAQ) [29] was used to assess frequency, duration and intensity of physical activity. Participants were asked to report the number of days in the previous week they had done each activity for more than 10 minutes at a time, and how long they would usually spend doing each activity. The leisure time physical activity (LTPA) domain was used in the analysis. Weekday and weekend sedentary behaviour over the previous week was also estimated using the IPAQ. Participants reported the average amount of time they had spent sitting on weekdays and weekend days during the previous week. This question has been shown to have acceptable reproducibility (one week test-retest reliability intraclass correlation range of 0.74–0.89) and comparative validity (rank correlation with one week accelerometer counts range of 0.20 – 0.51) [29]. In addition, participants' reported total time spent watching television, videos or DVDs when it was the main activity they were doing. This question has also been shown to have acceptable reproducibility (one week test-retest intraclass correlation coefficient 0.82) and comparative validity (rank correlation with three day sedentary behaviour log 0.3) [30].

The frequency of consumption of nine alcoholic beverages from the FFQ and their average alcohol concentration [31] was used to estimate the number of standard drinks (10 gram of alcohol) consumed per week. Responses of never or less than once per month were given a value of zero. Participants were classified as non-drinkers, drinkers who consume up to 14 drinks per week, or drinkers who consume more than 14 drinks per week. These groups are based on Australian alcohol guidelines for low-risk drinking [32].

### Analysis

Prevalence ratios estimated using log binomial regression or Poisson regression with robust standard errors [33] were used to summarise the associations of socio-economic and lifestyle variables, and moderate abdominal obesity with takeaway food consumption. Analyses were conducted separately for men and women. Covariates included in the adjusted analyses of obesity and takeaway food consumption were those that plausibly were causally related to the outcome or were markers of other factors causally related to the outcome, were not intermediate on the postulated pathway, and produced at least a 10% change in the parameter estimate for the study factor. They included age, LTPA, television viewing (log transformed) and employment status. Continuous variables were entered into the model as continuous covariates. Additional adjustments for marital status, education, smoking status, alcohol intake and other measures of physical activity did not materially alter the results. Interactions between takeaway food consumption and other covariates were assessed by including product terms as additional covariates.

Chi square analysis was used to examine the association between takeaway food consumption and meeting the dietary recommendations in the AGHE [26].

All statistical analyses were conducted with STATA software (version 9.2, 2007, Statacorp, College Station, Texas).

## Results

In total, 2,881 participants answered the dietary questionnaires. Nineteen participants did not answer the takeaway food question and were excluded from all analyses. The remaining 2,862 participants were included in the analysis of socio-demographic and lifestyle factors associated with takeaway food consumption. The dietary recommendation analysis excluded 78 women who were pregnant at the time of data collection because different dietary recommendations exist for pregnant women. A further 99 participants were excluded from the dietary recommendation analysis because they failed to provide responses to 10% or more of the FFQ items (n = 2,685 for analysis). The abdominal obesity analysis was restricted to clinic attendees who had anthropometric measurements and excluded pregnant women (n = 2,194 for analysis).

The socio-demographic characteristics and anthropometric measurements (for clinic attendees) of 2,862 participants (99.3% of questionnaire respondents) are shown in Table 1. The mean waist circumference (cm) was 89.5 (SD 10.6) for men and 78.2 (SD 11.4) for women. Men had a mean BMI of 26.5 (SD 4.2) kg/m^2^, while women had a mean BMI of 25.0 (SD 5.2) kg/m^2^.

**Table 1:** Socio-demographic and anthropometric characteristics of participants

	Men (n = 1277)*		Women (n = 1585)*	
	%	n	%	n
Age (mean, SD)	31.7	2.6	31.6	2.6
Married/living as married	66.9	854	72.2	1144
Education				
University	37.3	475	45.1	714
Vocational	35.7	455	26.0	412
School only	26.9	343	28.9	457
Occupation				
Professional/Manager	57.1	719	49.0	763
White collar	7.6	96	26.5	421
Blue collar	31.7	399	5.1	79
Not in workforce	3.6	45	19.5	303
BMI (kg/m^2^)^†^				
Normal (< 25 kg/m^2^)	38.4	409	62.0	701
Overweight (25 – 29.9 kg/m^2^)	45.4	483	23.8	269
Obese (≥ 30 kg/m^2^)	16.2	172	14.2	160
Waist circumference (cm)^†^				
Normal	72.4	771	65.9	744
Moderate abdominal obesity^‡^	27.6	294	34.1	385

* Sample sizes vary due to missing data (range 1,259 to 1,277 for men, 1,557 to 1,585 for women).

^† ^Anthropometric measurements in clinic attendees only and exclude pregnant women (men n = 1,065, women n = 1,129)

^‡ ^Moderate abdominal obesity was defined as ≥ 94 cm for men and ≥ 80 cm for women

While the study sample was derived from a nationally representative sample of children first measured in 1985, only one third participated in the follow-up in adulthood. Compared with the general Australian population of similar age (25–34 years) this study sample had a higher proportion of participants who were married or living as married (57% of men and 64% of women in the general population [34]), and a higher proportion of professionals/managers (40% of men and 38% of women in the general population [35]). The proportion of participants who were classified as overweight or obese (BMI ≥ 25 kg/m^2^) was similar to the general population (58% of men and 35% of women [36]).

### Takeaway food consumption

**Figure 1:**
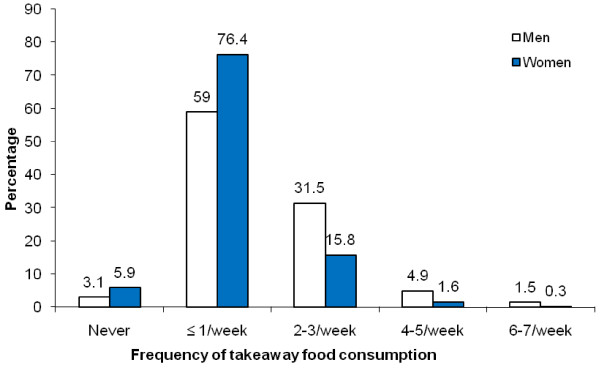
**Frequency of takeaway food consumption for men and women**. Difference between men (n = 1,277) and women (n = 1,585), P < 0.001.

The majority of participants (62.1% men and 82.3% women) ate takeaway once a week or less (Figure 1). Men consumed takeaway more frequently than women, with 37.9% of men and 17.7% of women eating takeaway at least twice a week (P < 0.001).

Our validation analysis showed takeaway food consumption from the short question in the FHQ was consistent with reported consumption of foods that are commonly eaten as takeaway food in the FFQ. Intake of the takeaway type foods was higher in participants who reported in the short question that they ate takeaway twice a week or more (52.8%, 404/765) than in participants who reported eating takeaway once a week or less (17.8%, 373/2097).

### Takeaway food consumption and socio-economic and lifestyle variables

Men who consumed takeaway at least twice a week were more likely to be single, younger, current smokers, to spend more time watching TV and to spend more time sitting (Additional File 1). Women who consumed takeaway at least twice a week were more likely to be single, in the workforce, and to spend more time watching TV and sitting.

### Achieving the dietary recommendations

The proportions of data that were missing were less than 10% in all food groups apart from lean meat and alternatives (37%). The proportions of missing data in each food group were not significantly different by takeaway food consumption, with the exception of lean meat; those consuming takeaway twice a week or more had more missing data (P = 0.043 for men, P = 0.033 for women).

Overall, compliance with the dietary recommendations was low, except for the lean meat and alternatives recommendation. Compliance was generally lower in participants who ate takeaway food more frequently (Table 2). Men who ate takeaway twice a week or more were significantly less likely to achieve the dietary recommendations for breads and cereals, vegetables, fruit, dairy, and extra foods. A similar result was found for women with those eating takeaway twice a week or more being significantly less likely to achieve the dietary recommendations for vegetables, fruit, dairy, lean meat and alternatives, and extra foods. Overall participants eating takeaway twice a week or more met fewer of the dietary recommendations (Table 3).

**Table 2:** Percentage of men and women achieving dietary recommendations for Australian adults by takeaway food consumption

Dietary recommendation*	Consuming takeaway < 2/week		Consuming takeaway ≥ 2/week		P-value
	%	n/N	%	n/N	
Men^†^					
Breads and cereals	5.3	41/774	2.6	12/462	0.023
Vegetables^‡^	8.7	67/773	5.0	23/460	0.017
Fruit	43.4	335/772	30.7	141/460	< 0.001
Dairy	41.3	320/774	32.9	152/462	0.003
Lean meats and alternatives	81.8	633/774	79.7	368/462	0.356
Extra foods (excluding takeaway)	38.0	294/774	28.4	131/462	0.001
					
Women^†^					
Breads and cereals	16.5	196/1186	18.3	48/263	0.499
Vegetables^‡^	14.2	168/1184	8.0	21/263	0.007
Fruit	51.8	613/1184	34.6	91/263	< 0.001
Dairy	39.5	468/1186	29.3	77/263	0.002
Lean meats and alternatives	89.1	1057/1186	84.4	222/263	0.032
Extra foods (excluding takeaway)	40.9	485/1186	30.0	79/263	0.001

*Daily Dietary Recommendations for Australian Adults aged 19–60 years recommend 6–12 servings (men) or 4–9 servings (women) of bread and cereals, 5 servings of vegetables, 2 servings of fruit, 2 servings of dairy, 1 serving of lean meat & alternatives, 0–3 (men) or 0–2 1/2 (women) servings of extra foods. Participants consuming at least the lower value for breads and cereals and not exceeding the upper limit for extra foods were classified as meeting the recommendation.

^†^Analysis excluded participants missing at least 10% of the FFQ (n = 99) and pregnant women (n = 78).

^‡ ^Participants classified as meeting the vegetable recommendation are consuming 4–5 serves/day not 5 as per the recommendation.

**Table 3:** Number of recommendations men and women were achieving by takeaway food consumption

Number of recommendations* achieved	Consuming takeaway < 2/week		Consuming takeaway ≥ 2/week		P-value
	%	n	%	n	
Men		n = 772		n = 460	
0	4.9	38	7.2	33	
1	22.8	176	32.6	150	
2	36.4	281	40.2	185	
3	23.1	178	14.6	67	
4-6	12.8	99	5.4	25	P < 0.001
Women		n = 1183		n = 263	
0	2.6	31	4.6	12	
1	18.7	221	29.7	78	
2	28.6	338	33.1	87	
3	29.4	348	23.2	61	
4-6	20.7	245	9.5	25	P < 0.001

*Daily Dietary Recommendations for Australian Adults aged 19–60 years recommend 6–12 servings (men) or 4–9 servings (women) of bread and cereals, 5 servings of vegetables, 2 servings of fruit, 2 servings of dairy, 1 serving of lean meat and alternatives, 0–3 (men) or 0–2 1/2 (women) servings of extra foods. Participants consuming at least the lower value for breads and cereals and not exceeding the upper limit for extra foods were classified as meeting the recommendation. Participants classified as meeting the vegetable recommendation are consuming 4–5 serves/day. Note: P-values calculated using Chi square analysis with groups 4–6 combined so cell values were all > 5.

When we repeated the fruit and vegetable analysis using daily intakes calculated from the FFQ, the intake of vegetables (men and women) and fruit (men only) was higher than with the short questions. This meant that a higher number of participants were classified as meeting these recommendations. However, the proportion meeting the recommendations remained significantly lower in participants who ate takeaway more frequently. This is consistent with the results using the short questions from the FHQ.

Milk in hot beverages was not included in the main analysis of the dairy food group because doing so could overestimate dairy intake for people who only add a small amount of milk to their hot drink. When we included milk consumed in hot beverages, we found a greater proportion of participants met the recommendations for dairy intake. For men, the difference between the takeaway food groups was no longer significant (71.0% consuming takeaway foods once a week or less versus 73.5% consuming takeaway food twice a week or more, P = 0.337), whereas, for women, those eating takeaway once a week or less remained more likely to meet the guidelines compared with those eating takeaway twice a week or more (78.4% versus 68.4%, respectively, P = 0.001).

### Factors associated with moderate abdominal obesity

Men with moderate abdominal obesity were more likely to be married (P < 0.005), older (P = 0.005) and watch more TV (P = 0.001). There was a non-linear trend for education where men with higher education were more likely to have moderate abdominal obesity (P < 0.001). Women with moderate abdominal obesity tended to be older (P = 0.024), less educated (P < 0.001), not in the workforce (P < 0.001), current smokers (P = 0.018), non-drinkers (P = 0.001), spend more time watching TV (P < 0.001), less physically active (P = 0.007), and to have more children (P = 0.002).

### Takeaway food consumption and moderate abdominal obesity

Men consuming takeaway at least twice per week were 33% more likely to have moderate abdominal obesity compared with men who ate takeaway less than twice a week (Table 4). This difference remained after adjusting for age, LTPA, TV viewing and employment status. Women consuming takeaway at least twice per week were 22% more likely to have moderate abdominal obesity compared with women consuming takeaway less than twice per week. This increased slightly to 25% after adjusting for age, LTPA, TV viewing and employment status. An interaction (P = 0.049) was found for women between smoking status and takeaway food consumption with the effect of takeaway consumption on waist circumference being strongest in never smokers (data not shown).

**Table 4:** Prevalence ratios of overweight and obesity for frequency of takeaway food consumption

	Frequency of takeaway food consumption	%	n/N	Unadjusted PR	95% CI	Adjusted PR*	95% CI
Men							
WC ≥ 94 cm	< 2/week	24.4	158/647	1.00		1.00	
	≥ 2/week	32.5	136/418	1.33	1.10, 1.62	1.31	1.07, 1.61
BMI ≥ 25 kg/m^2^	< 2/week	61.8	400/647	1.00		1.00	
	≥ 2/week	61.2	255/417	0.99	0.90, 1.10	0.98	0.88, 1.09
BMI ≥ 30 kg/m^2^	< 2/week	14.7	95/647	1.00		1.00	
	≥ 2/week	18.5	77/417	1.26	0.96, 1.65	1.21	0.90, 1.63
Women^†^							
WC ≥ 80 cm	< 2/week	32.7	297/909	1.00		1.00	
	≥ 2/week	40.0	88/220	1.22	1.02, 1.48	1.25	1.04, 1.50
BMI ≥ 25 kg/m^2^	< 2/week	36.5	332/910	1.00		1.00	
	≥ 2/week	44.1	97/220	1.21	1.02, 1.44	1.22	1.03, 1.45
BMI ≥ 30 kg/m^2^	< 2/week	13.3	121/910	1.00		1.00	
	≥ 2/week	17.7	39/220	1.33	0.96, 1.85	1.29	0.93, 1.80

PR = prevalence ratio, calculated using log binomial regression. WC = waist circumference.

*Adjusted for age, leisure time physical activity, TV viewing and employment status.

^†^Pregnant women (n = 78) were excluded from this analysis.

The adjustments for LTPA, TV viewing and employment status (men) reduced the coefficient of takeaway food consumption in the regression of waist circumference because those factors were negatively (LTPA) or positively (TV viewing, employment status of men) correlated with waist circumference. Adjusting for age and employment status (women) increased the coefficient of takeaway food consumption because those factors were negatively (employment status of women) or positively (age) correlated with waist circumference. In multivariable analysis, these four factors were significant predictors of consuming takeaway food at least twice a week.

Using BMI in place of waist circumference as the outcome variable, an association with takeaway food consumption was only found for men classified as being obese though this association was not statistically significant. In contrast, women eating takeaway food twice a week or more had a significantly higher prevalence of overweight and obesity.

## Discussion

We have shown takeaway food consumption is associated with a poorer diet quality and a higher prevalence of moderate abdominal obesity in young Australian adults. Different socio-economic and lifestyle factors are associated with a higher frequency of takeaway food consumption in men and women.

Differences in the methods used to ascertain takeaway and fast food consumption and the definition of takeaway or fast food used make it difficult to compare findings across studies. The frequency of takeaway food consumption in the current study was higher than that reported in a Mediterranean population (aged 24–75 years) where only 1.1% were consuming fast food at least twice per week but only hamburgers, cheese burgers, Big Macs and French fries were included as fast food [9]. A study in the USA reported 30% of men and 24% of women (aged 20 years and older) had consumed fast food on at least one of the two days studied using 24-hour diet recalls [4].

The socio-economic and lifestyle characteristics we found to be associated with higher frequency of takeaway food consumption were similar to those found in previous studies: younger age [5,8], being single [7,9] and watching more television [7]. However, to our knowledge this is the first study to report characteristics of takeaway food consumption separately for men and women. Being single and spending more time watching TV and sitting were associated with takeaway food consumption in both sexes. In men, being younger and a current smoker were also associated with takeaway consumption whereas in women, there was an association with employment status.

We found men consumed takeaway more frequently than women, which is consistent with some studies [6,37], but not others [8,9]. In contrast to a previous study [7] we found no significant association between takeaway food consumption and alcohol consumption in men or women.

Studies of socio-economic position and diet quality report that people of lower socio-economic status consume diets that are higher in energy dense foods such as takeaway foods [38]. However, our measures of socio-economic status (employment status and education) in this sample of young Australian adults do not support this. Participants who were not in the workforce were not high consumers of takeaway food, possibly because they could not afford to purchase it, and education had no association with takeaway food consumption. Previous studies investigating associations between income and takeaway food consumption have reported mixed results with some studies reporting participants with a high income to be the highest consumers [6,11], some showing participants with a low income to be the highest consumers [5], and yet others showing no association [15]. Education also shows mixed results with the majority of studies being consistent with our finding of no association [6,8,15], but others have reported positive associations with high education [9] or low education [7].

The number of participants achieving individual dietary recommendations was very low and lowest in participants who were eating takeaway food more frequently. This suggests takeaway food is not just an additional food item in an otherwise healthy diet but is associated with a number of other unhealthy eating behaviours, possibly by displacing healthier items from the diet. Our findings are similar to previous studies from the USA and Spain that report a higher frequency of takeaway or fast food consumption is associated with a lower intake of fruit, vegetables and dairy [5,7-9]. A higher frequency of takeaway food consumption was associated with a lower intake of breads and cereals in men and a lower intake of lean meats and alternatives in women. Overall participants eating takeaway food more frequently met fewer of the dietary recommendations. This supports the previous study in Spain that examined overall diet quality, where participants eating fast food at least twice per week had the lowest adherence to the Healthy Eating Index and the Mediterranean Diet Score [9].

This is the first study to show that young adults eating takeaway more frequently have a somewhat higher prevalence of moderate abdominal obesity as measured by waist circumference. Women eating takeaway food twice a week or more had a higher prevalence of being overweight or obese as defined by a BMI ≥ 25 kg/m^2 ^and this association remained significant after adjusting for covariates. However, in men, an association was only seen at the higher level of BMI (≥ 30 kg/m^2^) and the association was not significant. While we were unable to adjust for energy intake, because this was not available from the FFQ, we did take into account key determinants of energy intake by stratifying the analysis by sex and adjusting for age and physical activity levels. Due to the cross-sectional analysis, we cannot be certain of the direction of a causal relationship between takeaway food consumption and abdominal obesity. Although our study sample comes from a cohort study, longitudinal analysis is not possible because comparable dietary data were not collected in childhood. Previous studies have found an association between takeaway and fast food consumption and BMI [9] and changes in weight over time [5,7,12,39].

There are several limitations with the dietary recommendation analysis. First, the response option for the vegetable question combined four and five serves per day, and the proportion meeting the vegetable recommendation (at least five daily serves) is likely to be lower than that reported here. In addition, compliance with the lean meat and alternatives recommendation may be overestimated due to the large number of items included in this variable. However, previous national data show consumption of meat and alternatives is high in Australian adults [40]. Second, although we excluded from the analysis participants who had not adequately completed the questionnaire (those that failed to complete > 90% of the FFQ), we were left with occasional non-responses to items by the remaining respondents. These were assigned a value of zero on the grounds that a non-response indicated the respondent did not eat that food. However, some of these missing items may have been overlooked by the respondent. If so, this would have resulted in under-estimation of the proportions of respondents meeting the dietary recommendations. It is reassuring that this measurement error did not appear to be differential between the two takeaway food groups, with the exception of the lean meat and alternatives food group. Third, the guidelines recommend consuming wholegrain breads and cereals; apart from bread, the FFQ did not distinguish between wholegrain and non-wholegrain items. Fourth, components of mixed dishes were not included as items in the food groups and may be under-estimated. Mixed dishes are generally difficult to assess using FFQs [41].

A strength of this study was that we asked about *usual *takeaway food consumption and, in addition to food available from the main fast food chains (McDonalds, Pizza Hut, KFC etc), our takeaway food variable included other popular takeaway food options such as Indian, Thai and Chinese foods. Furthermore, this is the first study to report associations of takeaway food consumption with lifestyle factors separately for men and women. We did this because we were interested in examining potential sex differences in takeaway food consumption to better understand the predictors of this eating behaviour. Other strengths include the use of a FFQ that has been used in previous national surveys, and examining overall diet quality, which has been done in only one previous study.

## Conclusion

In this large nationwide study of young Australian adults we found participants consuming takeaway food at least twice per week met fewer of the dietary recommendations and had a modestly higher prevalence of moderate abdominal obesity compared with participants consuming takeaway once a week or less. Initiatives to reduce takeaway food consumption or to promote healthier takeaway food options have the potential to improve diet quality and prevent obesity.

## Abbreviations

FFQ: food frequency questionnaire; FHQ: food habits questionnaire; IPAQ: international physical activity questionnaire; LTPA: leisure time physical activity; PR: prevalence ratio; WC: waist circumference.

## Competing interests

The authors declare that they have no competing interests.

## Authors' contributions

KS performed the statistical analysis and drafted the manuscript. SM provided nutritional advice and helped draft the manuscript. SG provided analytical and interpretive advice and helped draft the manuscript. LB provided statistical support and critical revision of the manuscript. TD was involved in conceptualisation of the study and provided critical revision of the manuscript. AV was involved in the conceptualisation of the study, acquisition of data and helped draft the manuscript. All authors read and approved the final manuscript.

## Appendix 1 – Items from food frequency questionnaire that were included in daily equivalents

### Breads and cereals

White bread, toast or rolls

Wholemeal/mixed grain bread, toast or rolls

English muffin, bagel or crumpet

Flat bread (e.g. pita, chapatti)

Dry or savoury biscuits, crispbread, crackers

Muesli

Cooked porridge

Breakfast cereal

Rice (white or brown)

Pasta (including filled), noodles

### Dairy

Flavoured milk drink (e.g. milkshake, iced coffee, hot chocolate)

Milk as a drink

Milk added to breakfast cereal

Yoghurt, plain or flavoured (including fromage frais)

Cheddar and other cheeses

Soy milk

### Lean meat, fish, eggs

* Mince dishes (e.g. rissoles, meatloaf)

* Mixed dishes with beef, veal, lamb, pork (e.g. casserole, stir fry)

* Beef, veal – roast, chop or steak

* Lamb – roast, chop

* Pork – roast, chop

* Mixed dishes with chicken, duck, turkey (e.g. casserole, stir-fry)

* Chicken, turkey, duck – roast, steamed or barbequed

Canned fish (e.g. tuna, salmon, sardines)

Fresh fish – steamed, baked, grilled

Frozen fish – steamed, baked, grilled

Mussels/oysters

Lobster/crayfish/yabbies

Calamari/squid

Prawns

Other seafood

Egg

Almonds, walnuts, hazelnuts

Cashews

Coconuts

Peanuts

Pistachio

Seeds – pumpkin, sesame, pine nuts, tahini

Other nuts, seeds

Baked beans

Other beans, lentils

* These items were only included as lean meat if participant indicated in the food habits questionnaire that fat was usually trimmed from their meat either before or after cooking.

### Extra foods – excluding takeaway

Cream or sour cream

Ice cream

Cakes, sweet muffins, scones or pikelets

Sweet pies or sweet pastries

Other pudding or desserts

Plain, sweet biscuits

Cream, chocolate biscuits

Chocolate (including chocolate bars e.g. Mars bar™)

Other confectionary

Potato chips, corn chips, Twisties™ etc

Oil and vinegar dressing

Mayonnaise or other creamy dressings

Jam, marmalade, syrup or honey

Creamy dips and spreads

Fruit juice drink or fruit cordial

Cordial

Soft drinks (including flavoured mineral water)

Light beer

Medium strength beer

Full strength beer

Red wine

White wine or champagne/sparkling wine

Wine cooler

Spirit-based mixed drinks (e.g. Lemon Ruski™)

Sherry/port/fortified wines

Spirits, liquers

Other alcoholic drinks (e.g. cider)

### Fruit and vegetables

Note: Daily equivalents of fruits and vegetables were obtained from short questions in the food habits questionnaire.

### Additional analysis

#### Vegetables

Green/mixed salad (including lettuce, tomato etc) in a sandwich

Green/mixed salad (including lettuce, tomato etc) as a side-salad with a main meal

Stir-fried or mixed vegetables

Vegetable casserole

Sweet potato

Pumpkin

Peas (including snow peas)

Green beans

Silverbeet/spinach

Broccoli

Cauliflower

Brussel sprouts, cabbage, coleslaw

Carrots

Mushrooms

Capsicum

Sweetcorn, corn on the cob

Zucchini, eggplant, squash

Cucumber

Tomatoes (except when in a 'mixed salad')

Lettuce (except when in a 'mixed salad')

Celery (except when in a 'mixed salad')

Onion or leek

Soy beans, tofu

Baked beans

Other beans, lentils

#### Fruits

Fruits – dried frozen, canned

Fresh fruit salad

Apple or pear

Orange, mandarin, grapefruit

Banana

Peach, nectarine, plum or apricot

Mango or paw paw

Pineapple

Grapes or berries

Melon (watermelon, rockmelon or honeydew melon)

Other fruit not listed

## Supplementary Material

Additional file 1 **Socio-economic and lifestyle factors associated with consuming takeaway food at least twice a week.** Click here for file
