# Supplementary material for: Takeaway food consumption and its associations with diet quality and abdominal obesity: a cross-sectional study of young adults
Source: Int J Behav Nutr Phys Act. 2009 May 28;6:29. doi: 10.1186/1479-5868-6-29 (PMC2694758; doi:10.1186/1479-5868-6-29)
Supplement: Additional file 1 — Socio-economic and lifestyle factors associated with consuming takeaway food at least twice a week. [file 1479-5868-6-29-S1.docx]

### Additional File 1 - Socio-economic and lifestyle factors associated with consuming takeaway food at least twice a week

|  | | Men | | | | Women | | | |
| --- | --- | --- | --- | --- | --- | --- | --- | --- | --- |
| Socioeconomic/lifestyle factor | | % | n/N* | PR | 95% CI | % | n/N* | PR | 95% CI |
| Marital Status | |  |  |  |  |  |  |  |  |
|  | Single | 46.3 | 196/423 | 1.00 |  | 20.7 | 91/440 | 1.00 |  |
|  | Married/living as married | 33.7 | 288/854 | 0.73 | 0.63, 0.84 | 16.6 | 190/1144 | 0.80 | 0.64, 1.01 |
|  | *Trend* |  |  | *P<0.001* |  |  |  | *P=0.055* |  |
| Age | |  |  |  |  |  |  |  |  |
|  | 26-29 years | 47.1 | 136/289 | 1.00 |  | 19.8 | 82/415 | 1.00 |  |
|  | 30-33 years | 38.4 | 232/604 | 0.82 | 0.70, 0.96 | 18.0 | 131/729 | 0.91 | 0.71, 1.17 |
|  | 34-36 years | 30.2 | 116/384 | 0.64 | 0.53, 0.78 | 15.4 | 68/441 | 0.78 | 0.58, 1.05 |
|  | *Trend* |  |  | *P<0.001* |  |  |  | *P=0.096* |  |
| Education | |  |  |  |  |  |  |  |  |
|  | University | 36.0 | 171/475 | 1.00 |  | 17.1 | 122/714 | 1.00 |  |
|  | Vocational | 39.3 | 179/455 | 1.09 | 0.93, 1.29 | 17.2 | 71/412 | 1.01 | 0.77, 1.32 |
|  | School only | 38.2 | 131/343 | 1.06 | 0.89, 1.27 | 19.3 | 88/457 | 1.13 | 0.88, 1.44 |
|  | *Trend* |  |  | *P=0.471* |  |  |  | *P=0.364* |  |
| Employment status | |  |  |  |  |  |  |  |  |
|  | Not in workforce | 22.2 | 10/45 | 1.00 |  | 10.6 | 32/303 | 1.00 |  |
|  | In workforce | 38.1 | 462/1214 | 1.71 | 0.99, 2.97 | 19.5 | 245/1254 | 1.85 | 1.31, 2.62 |
|  | *Trend* |  |  | *P=0.056* |  |  |  | *P<0.001* |  |
| Smoking status | |  |  |  |  |  |  |  |  |
|  | Never smoked | 35.9 | 282/785 | 1.00 |  | 16.9 | 158/933 | 1.00 |  |
|  | Former smoker | 37.6 | 71/189 | 1.05 | 0.85, 1.29 | 19.2 | 60/312 | 1.14 | 0.87, 1.48 |
|  | Current smoker | 46.4 | 108/233 | 1.29 | 1.09, 1.52 | 20.6 | 51/248 | 1.21 | 0.92, 1.61 |
|  | *Trend* |  |  | *P=0.005* |  |  |  | *P=0.144* |  |
| Alcohol consumption | |  |  |  |  |  |  |  |  |
|  | Non-drinker | 35.7 | 41/115 | 1.00 |  | 15.7 | 50/318 | 1.00 |  |
|  | ≤ 14 drinks/week | 39.1 | 375/958 | 1.10 | 0.85, 1.42 | 17.7 | 206/1167 | 1.12 | 0.85, 1.49 |
|  | > 14 drinks/week | 33.0 | 66/200 | 0.93 | 0.68, 1.27 | 25.5 | 25/98 | 1.62 | 1.06, 2.48 |
|  | *Trend* |  |  | *P=0.413* |  |  |  | *P=0.058* |  |
| TV viewing | |  |  |  |  |  |  |  |  |
|  | < 8 hours/week | 35.0 | 111/317 | 1.00 |  | 13.7 | 73/532 | 1.00 |  |
|  | 8-14 hours/week | 36.0 | 128/356 | 1.03 | 0.84, 1.26 | 15.8 | 80/506 | 1.15 | 0.86, 1.54 |
|  | 15-21 hours/week | 35.9 | 98/273 | 1.03 | 0.82, 1.28 | 24.3 | 67/276 | 1.77 | 1.31, 2.38 |
|  | > 21 hours/week | 48.1 | 101/210 | 1.37 | 1.12, 1.69 | 27.0 | 47/174 | 1.97 | 1.42, 2.72 |
|  | *Trend* |  |  | *P=0.006* |  |  |  | *P=<0.001* |  |
| Sitting | |  |  |  |  |  |  |  |  |
|  | < 20 hours/week | 31.7 | 66/208 | 1.00 |  | 12.6 | 39/310 | 1.00 |  |
|  | 20-40 hours/week | 36.9 | 152/412 | 1.16 | 0.92, 1.47 | 15.8 | 92/581 | 1.26 | 0.89, 1.78 |
|  | 41-60 hours/week | 40.4 | 128/317 | 1.27 | 1.00, 1.62 | 22.7 | 85/375 | 1.80 | 1.27, 2.55 |
|  | > 60 hours/week | 41.0 | 91/222 | 1.29 | 1.00, 1.67 | 23.9 | 52/218 | 1.90 | 1.30, 2.77 |
|  | *Trend* |  |  | *P=0.032* |  |  |  | *P<0.001* |  |
| Leisure time physical activity | |  |  |  |  |  |  |  |  |
|  | 0-1 hours/week | 39.4 | 194/493 | 1.00 |  | 19.7 | 124/630 | 1.00 |  |
|  | 1.1 – 3 hours/week | 34.2 | 97/284 | 0.87 | 0.71, 1.06 | 17.4 | 72/414 | 0.88 | 0.68, 1.15 |
|  | 3.1 – 5 hours/week | 42.7 | 73/171 | 1.08 | 0.88, 1.33 | 19.3 | 47/243 | 0.98 | 0.73, 1.33 |
|  | > 5 hours/week | 34.1 | 74/217 | 0.87 | 0.70, 1.07 | 12.9 | 28/217 | 0.66 | 0.45, 0.96 |
|  | *Trend* |  |  | *P=0.426* |  |  |  | *P=0.069* |  |

PR = prevalence ratio, calculated using log binomial regression.

* Sample sizes vary due to missing data (range 1,156 to 1,277 for men, 1,488 to 1,585 for women).
